# Efficacy of a mesenchymal stem cell loaded surgical mesh for tendon repair in rats

**DOI:** 10.1186/1479-5876-12-110

**Published:** 2014-05-02

**Authors:** Lew C Schon, Nicholas Gill, Margaret Thorpe, Joel Davis, Joshua Nadaud, Jooyoung Kim, Jeremy Molligan, Zijun Zhang

**Affiliations:** 1 Department of Orthopaedic Surgery, MedStar Union Memorial Hospital, 3333 North Calvert Street, Johnston Professional Building, Suite 400, Baltimore, MD 21218, USA; 2 Orthobiologic Laboratory, Medstar Union Memorial Hospital, Baltimore MD, USA; 3 University of Cincinnati School of Medicine, Cincinnati OH, USA; 4 Mid County Orthopaedic Surgery & Sports Medicine, St. Louis MO, USA

**Keywords:** Mesenchymal stem cells, Tendon, Achilles, Surgical mesh, Rats

## Abstract

**Objectives:**

The purpose of this study was to investigate the efficacy of a composite surgical mesh for delivery of mesenchymal stem cells (MSCs) in tendon repair.

**Methods:**

The MSC-loaded mesh composed of a piece of conventional surgical mesh and a layer of scaffold, which supported MSC-embedded alginate gel. A 3-mm defect was surgically created at the Achilles tendon-gastrocnemius/soleus junction in 30 rats. The tendon defects were repaired with either 1) MSC-loaded mesh; or 2) surgical mesh only; or 3) routine surgical suture. Repaired tendons were harvested at days 6 and 14 for histology, which was scored on the bases of collagen organization, vascularity and cellularity, and immunohistochemisty of types I and III collagen.

**Results:**

In comparison with the other two repair types, at day 6, the MSC-loaded mesh significantly improved the quality of the repaired tendons with dense and parallel collagen bundles, reduced vascularity and increased type I collagen. At day 14, the MSC-loaded mesh repaired tendons had better collagen formation and organization.

**Conclusion:**

The MSC-loaded mesh enhanced early tendon healing, particularly the quality of collagen bundles. Application of the MSC-loaded mesh, as a new device and MSC delivery vehicle, may benefit to early functional recovery of the ruptured tendon.

## Introduction

Tendon injury is one of the most common musculoskeletal conditions. Surgical repair of the ruptured tendon is the standard of care. The long-term outcomes of the surgery, however, vary greatly. In general, the repair is inefficient: tendon heals slowly (8–12 weeks) [1]. After surgical repair, in most of the cases, the tendon heals not by a regenerative process (intrinsic healing) but rather by the formation of scar tissue (extrinsic healing) [2]. The inferior properties of the repairing tissue may cause significant dysfunction and even disability [3,4]. Clinically, there is great demand to improve the surgical techniques and efficiency of tendon repair.

Tendon rupture can occur at the muscle-tendon junction, the bone-tendon junction and the middle portion of the tendon. Each type of the tendon injury has different healing processes [5] and presents unique challenges to repair. Suture is routinely used for tendon repair to provide the essential stability for healing [6], but muscle is a poor retainer of suture. To overcome this difficulty, surgical mesh has been used to patch the ruptured muscle-tendon junction to enforce the repair with broadly distributed tension in the muscle. Achilles tendon ruptures are relatively common, with an estimated rate of 18 per 100,000 people [6]. Approximately, 12% of Achilles tendon injuries are ruptures at the muscle-tendon junction [7].

Tenocytes that reside in the tendon are fully differentiated and therefore have little capability of regeneration [8]. Mesenchymal stem cells (MSCs) from the stroma of bone marrow and other tissues are multipotent and capable of forming bone, cartilage and other connective tissues [9,10]. Although the regulation and milestone markers of tenogenic differentiation are still undefined [3], MSCs differentiating into tenocytes and forming tendon tissue have been demonstrated [11-14]. More impressively, implanted alone or with other biomaterials, MSCs formed tendon-like tissue *in vivo*[15]. Clinical applications of MSCs may fundamentally change the process of tendon repair. It has been found that the MSC-differentiated tendocytes produce largely type I collagen but not type III collagen [16], which is increased in the early stage of scar-like tendon repair. Supplementation of MSCs to tendon repair has been largely beneficial [17,18]. For example, MSC-loaded collagen gel significantly reinforced the mechanical strengthen of repaired patellar tendon [19,20].

MSCs have the potential to modulate the process of tendon repair or regenerate neo-tendon with improved tissue structure, collagen organization, and matrix composition and thus the clinical outcomes. This study was designed to investigate the efficacy of MSC-loaded surgical mesh in tendon healing at the Achilles tendon-gastrocnemius/soleus junction in rats. The repaired Achilles tendons were examined with histology and immunohistochemistry of types I and III collagen for evaluation of the quantity of tendon repair.

## Materials and methods

### Design of MSC-loaded surgical mesh

The MSC-loaded surgical mesh was an integrated biologically active composite structure consisting of surgical mesh, polymer scaffold and MSC embedded hydrogel. The knitted surgical mesh was made of polypropylene (PPM1 Retain Mesh; Lot # 10-001253-1015, Biomedical Structures, Warwick, RI), which is commonly used in surgery for tissue repair. The mesh formed a broad base for other layers to rest on. During tendon repair, the mesh was used to patch over the injury site and provided the necessary mechanical strength to bridge the ruptured tendon. In this study, the dimensions of the mesh were 17 mm x 7 mm.

**Figure 1:**
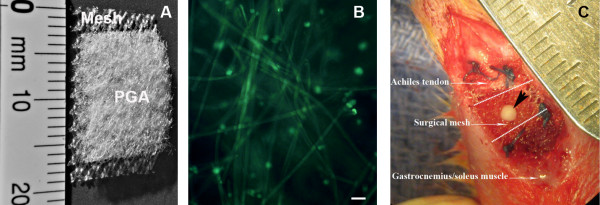
**Design and application of MSC-loaded surgical mesh. A**: Composition of MSC-loading mesh. **B**: Distribution of MSCs in the PGA-based scaffold. MSCs (green) were labeled with fluorescent dye SYTO®10. PGA fibers also showed green in auto-fluorescence (bar = 50 μm). **C**: The MSC-loaded mesh was used to repair the 3-mm defect (between the dot lines) created at the junction of Achilles tendon and gastrocnemius/soleus. The portion of scaffold/MSCs was inserted into the defect and surgical mesh was sutured to the surface of muscle and tendon (black arrowhead indicates a knot of surgical suture used to tie the scaffold and surgical mesh together. The suture was melted after autoclave).

The second component of the MSC-loaded mesh was a layer of non-woven scaffold, 4 x 4 x 2 mm^3^, made of polyglycolic acid (PGA, 60 mg/ml in density, Lot # 10-001253A-1015, Biomedical Structures). The scaffold was attached to the center of the surgical mesh with one knot using a 4–0 monofilament suture. The purpose of the PGA scaffold was to support and attach the hydrogel component to the mesh (Figure 1A).

### Preparation of the MSC embedded alginate hydrogel

For isolation of MSCs, human bone marrow samples were collected during orthopaedic surgery performed at MedStar Union Memorial Hospital, Baltimore, MD (approved by the Institutional Review Board of MedStar Health Research Institute). The bone marrow was diluted at a 1:1 ratio with phosphate buffered saline (PBS) with 2% fetal bovine serum (FBS) and then layered on top of the Ficoll-Paque™ PLUS density gradient medium (STEMCELL Technologies, Vancouver, Canada) in a 50-ml conical tube and centrifuged for 30 minutes at 400x g. The mononuclear cell layer at the plasma-Ficoll interface was removed and the mononuclear cells were washed once with PSB containing 2% FBS before being plated in tissue culture flasks at a density of 4,000 cells per cm^2^. The isolated MSCs were cultured in MesenCult® MSC Basal Medium supplemented with MesenCult® Mesenchymal Stem Cell Stimulatory Supplements (STEMCELL Technologies) at 37°C in a humidified incubator with 5% CO_2_ in air. The adherent MSCs were passaged at 60-80% confluence. At passage 2, 1x10^6^ MSCs were resuspended in 50 μl culture medium and then mixed with 50 μl 2% alginate. Of the alginate mixture, 40 μl (containing 4x10^5^ MSCs) was added onto the PGA scaffold and alginate polymerization was subsequently initiated with CaCl_2_ (100 mM). Finally, MSCs were embedded within alginate gel, which was attached to the surgical mesh via PGA scaffold.

Samples of the surgical mesh constructs were incubated with SYTO®10 (Life Technologies, Grand Island, NY), which is a highly membrane-permeable green fluorescent nucleic acid stain for viable cells, for MSC density and distribution in the scaffold. Examined under a fluorescent microscope, MSCs evenly distributed within alginate gel (Figure 1B).

### MSC-loaded mesh for tendon repair in rats

The MSC-loaded surgical mesh was used immediately in a rat model of tendon repair. A total of 30 Sprague-Dawley rats, male, body weight 250–300 g, were used for this study (approved by the Institutional Animal Care and Use Committee, MedStar Health Research Institute). The rats were anesthetized by intraperitoneal injection of Nembutal (pentobarbital, 0.05 mg/g). The right hind-limbs of the rats were shaved and prepared for surgery. A longitudinal midline incision was made on the lower limb to expose the Achilles tendon. The tendon was completely severed at the Achilles tendon-gastrocnemius/soleus junction. The muscle was transected 3 mm proximally to create a defect between the tendon and muscle tissue. According to the repair methods, rats were divided into three study groups: 1) repair using the composite surgical mesh loaded with MSCs (group M + S); 2) repair using the composite surgical mesh, without loading of MSCs (group M); or repair using suture only (group Sut). For animals receiving one of the two surgical mesh types, the mesh was applied so as to cover the created muscle-tendon defect. With the hydrogel/PGA scaffold filled in the defect, the mesh on the top was sutured to the Achilles tendon and the gastrocnemius/soleus muscle at either end of the defect (Figure 1C). For animals receiving suture repair, the Achilles tendon and muscle ends were loosely approximated through a figure-of-eight stitch with three knots; prior to tying the knots, an instrument 3 mm in width was placed between the tendon and muscle ends to preserve the 3-mm defect. After repaired the tendon, the wound was closed with 4–0 monofilament suture in a subcutaneous fashion. The operated limbs were not immobilized. Rats were allowed to access food and water *ad lib*.

In each group, 5 rats were euthanized at 6 and 14 days after repair. Tissues around the tendon defect were dissected *en bloc* and fixed with 10% buffered formalin. After embedded in paraffin, tissue samples were sectioned for histology and immunohistochemistry. Tissue sections (5 μm) were collected at 10 intervals throughout the width of the repair site in the longitudinal plane and numbered for better comparisons among the three groups.

Ten sections from each tendon sample were chosen at random and stained with hematoxylin and eosin (H&E). Picrosirius Red staining for collagen was also performed. The histology of tendon repair were evaluated by three scorers blinded to the repair methods used and scored on the bases of collagen organization, vascularity and cellularity according to a modified tendon histological scoring system [21,22]. Dense/clearly defined/parallel collagen bundles oriented tangentally, a sparse network of small arteries oriented parallel to the collagen fibers in thin/fibrous septa between the bundles, and an even/sparse distribution of cells between collagen bundles are characteristics of a normal tendon, which would have the lowest score (see Table 1 for details). The scores from the ten slides of each tendon were averaged.

**Table 1:** Histological score of tendon repair

**Score**	**Collagen**	**Angiogenesis**	**Cellularity**
0	dense, clearly defined parallel collagen bundles oriented tangentally	a sparse network of small arteries oriented parallel to the collagen fibers in thin, fibrous septa between the bundles	fairly even, sparse distribution of cells with thin wavy nuclei located between collagen bundles
1	less than 25% of collagen bundles have a diffuse structure with blurring of individual bundles	less than 25% irregular vascularization: increased capillaries	less than 25% abnormal increased cellularity with rounded nuclei oriented in rows
2	less than 50% of collagen bundles have a diffuse structure with blurring of individual bundles	less than 50% irregular vascularization: increased capillaries; groups of thick-walled vessels distributed unevenly in the hypercellular tendon	less than 50% abnormal increased cellularity with rounded nuclei oriented in rows
3	more than 50% of collagen bundles have a diffuse structure with blurring of individual bundles	more than 50% irregular vascularization: increased capillaries; groups of thick-walled vessels distributed unevenly in the hypercellular tendon; proliferating vessels are nodular and may be perpendicular to the collagen bundles	more than 50% abnormal increased cellularity with rounded nuclei oriented in rows

Immunohistochemistry for types I and III collagen was performed on the sections of repaired tendons that were collected at days 6 and 14 after surgery in all three study groups. After antigen retrieval (citric acid/EDTA buffer, pH 6), tissue sections were blocked with serum and hydrogen peroxide, before incubated with a mouse antibody of type III collagen (1:100 dilution, Sigma-Aldrich Co, St. Louis, MO) or goat antibody of type I collagen (1:100 dilution, Santa Cruz Biotechnology, Dallas, TX) overnight. Types I or III collagen was detected with either rabbit anti-goat or rabbit anti-mouse secondary antibody conjugated with horse radish peroxidase and substrate 3, 3’-diaminobenzidine (DAB) was used for colorimetric detection. Negative controls were performed by replacing primary antibody with normal mouse IgG or normal goat serum. Immunohistochemistry for each group was performed in triplicate. Immunohistochemical staining for type I or III collagen was carried out at the same time to reduce variables might be introduced during the procedure. All the tissue sections were imaged under the same microscope with the same settings in the same day. On each image, in a computer defined area, the staining area (predefined pixel units) and intensity (average grayscale) were measured using ImageJ (NIH).

#### Statistical analyses

Data were expressed as mean ± standard deviation. The overall histological scores of tendon healing and scores of sub-categories at days 6 and 14 were analyzed across the three study groups using two-way ANOVA, followed with Tukey’s *post hoc* analysis. P < 0.05 was set as statistically significant.

Similarly, the staining area and intensity of types I and III collagen among the three groups at different time-points were analyzed. In addition, type III collagen: type I collagen ratio in staining area and intensity of the same group at the same time point was calculated and compared among M + S, M and Sut groups at days 6 and 14.

## Results

All rats survived from the surgery and through the follow-up. No infection was observed at the surgical site. At the time of tissue collection, tendon defect was repaired grossly in all three study groups.

**Figure 2:**
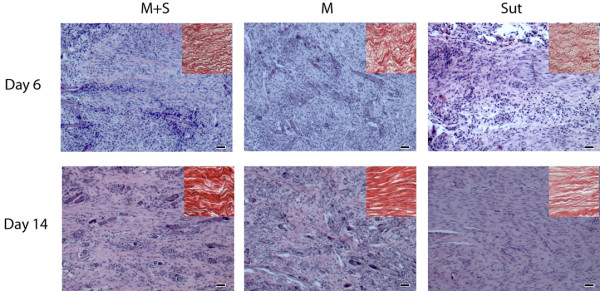
**Histology of repaired tendons.** At day 6, there was more extracellular matrix deposited in group M + S than in group M. Furthermore, the matrix appeared more organized and parallel with the long axis of the tendon. By day 14, there were more fibroblastic cells and increased matrix deposition in all three groups. The orientations of nuclei indicated that, in areas, cells in group M + S arranged in line with the long axis of the tendon and that was not seen in group M. Matrix area and staining density in group Sut were much less than groups M + S and M (H&E staining, inserts are Picrosirius Red staining; bar = 50 μm).

**Figure 3:**
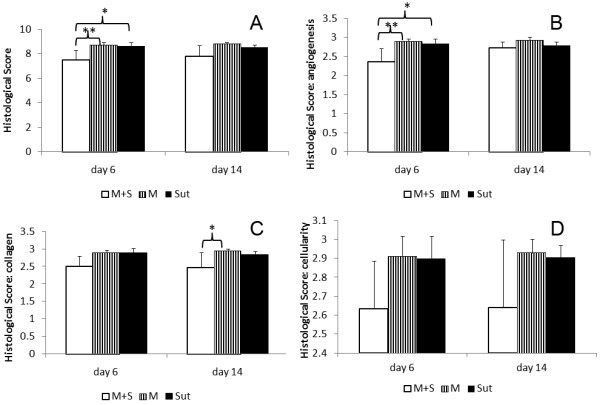
**Quantification of tendon healing. A**: The overall scores of the repaired tendons in three study groups. At day 6, tendon repair was significantly improved in group M + S. **B**: The angiogenesis scores of the repaired tendons. At day 6, angiogenesis in group M + S was significantly less than groups M and Sut. **C**: The collagen scores of the repaired tendons. The scores of collagen bundles in group M + S showed significant improvement at day 14. **D**: No differences were found among groups in cellularity score (*p < 0.05; **p < 0.001).

At day 6, histology showed that fibroblastic cells filled in the tendon defects in all three groups. Although cell density was high in both M and M + S groups, there was no inflammatory response around PGA fibers. Compared with M and Sut groups, there was more matrix deposition in M + S group (Figure 2) and the matrix appeared in an organized pattern that run in parallel along the tendon (insert). The modified tendon repair score of M + S group was significantly improved over the M and Sut groups (p < 0.05; Figure 3A). When the scores were further analyzed at subcategories, it appeared that reduced vascularity in M + S group contributed most to the overall quality of the repaired tendon in M + S group (Figure 3B).

By day 14, there were significant reduction of cellularity and increased deposition of extracellular matrix in the repaired tendon in all three groups (Figure 2). Compared with M and M + S groups, cells in Sut group were uniformly fibroblastic-like but deposited much less matrix. While matrix and cells in M group were in a chaotic or random pattern, much of the matrix and cells in M + S group aligned in accordance with the orientation of the tendon. Histomorphometrically, the modified tendon repair score of M + S group was improved over both M and Sut groups at day 14, but this was not statistically significant (Figure 3A). It was noticed that, under the subcategory of collagen, M + S group was significantly improved at day 14, compared with M and Sut groups (p < 0.05; Figure 3C).

**Figure 4:**
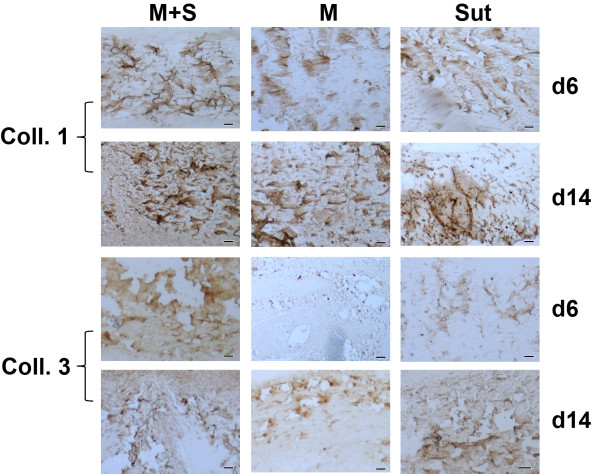
**Immunohistochemistry of types I and III collagen.** In general, type I collagen was stained in larger fibers, while type III collagen was detected on finer fibers in the repaired tendons. The distribution of types I and III collagen was very similar among the groups, except of noticeable weak staining of type III collagen in the M group at day 6 (bar = 50 μm).

**Figure 5:**
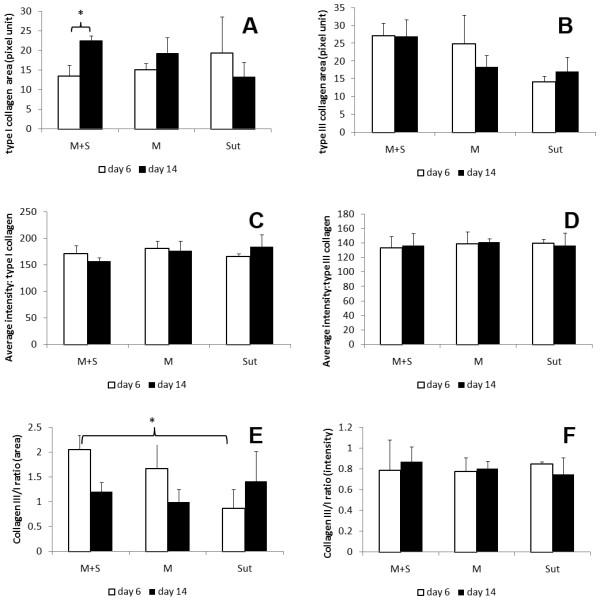
**Quantification of immunohistochemistry of types I and III collagen in tendon repair.** The staining of types I and III collagen was quantified in 1) staining area **(A and C)** and 2) staining intensity **(B and D)**. In M + S group, the area of type I collagen was significantly increased from day 6 to day 14. The staining area of type III collagen was indifferent among the three groups. The area ratio of type III collagen over type I collagen in M + S group was greater than in the Sut group at day 6 **(E)**. No differences were detected in average intensity of types I and III collagen, individually and the ratio **(F)**, among the three groups.

Immunohistochemistry demonstrated similar distribution patterns of types I and III collagen among the three groups (Figure 4). At the same time point, type I collagen staining area and intensity were not statistically different among the three groups in (Figure 5A and B). The area of type I collagen staining increased from day 6 to day 14 in the M + S groups (p < 0.05), but this was not in M and Sut groups.

Neither the area nor the average intensity of Type III collagen was significantly different among the three groups at both days 6 and 14 (Figure 5C and D).

The staining area ratio of type III collagen over type I collagen in the M + S group was the highest, but it was only statistically different between M + S and Sut groups (Figure 5E). There were interesting trends of the area ratio of type III collagen vs. type I collagen, although they were not statistically significant. The ratio was reduced from day 6 to day 14 in the M + S group (p = 0.06) and M group, but increased in the Sut group.

The intensity ratio of type III collagen over type I collagen showed not statistically different among M + S, M and Sut groups at both day 6 and day 14 (Figure 5F).

## Discussion

Surgical mesh is commonly used for repairing ruptures at the muscle-tendon junction. In the current study, the composite mesh was designed to not only bridge the defect between Achilles tendon and the gastrocnemius/soleus muscle but also carry MSCs to the site of tissue repair. The MSC-loaded mesh consisted of multiple components. All the materials making up the composite mesh are commonly used in the surgery and biomedical research [23,24]. The design of the composite mesh emphasized its practicability during a surgical procedure and versatility to varied tendon pathology and other clinical situations. The surgical mesh and scaffold can be tailored to any size and shape to adapt the gap of tendon rupture. The surgical mesh and scaffold were stitched together with surgical sutures. This gives surgeons another layer of flexibility to arrange and secure the scaffold to the mesh as needed on an individual basis. The conventional surgical mesh restored the mechanical continuity of the ruptured tendon and might provide a physical environment that stimulates tenogenesis of MSCs [25-27]. The scaffold was made of PGA, which is biodegradable and has been used for tendon repair [8]. In this MSC-loaded mesh, a layer of scaffold ensured uniform and 3-dimensional MSC distribution, which was confirmed by staining MSCs in the composite mesh. While injection of MSCs to the injury site of the tendon is convenient to apply [17,28], it is the advantage of the MSC-loaded mesh being able to distribute MSCs evenly throughout the tendon defect. This could be particularly important when MSCs are used to repair large tendons/ligaments, such as Achilles tendon and patellar ligament. Alginate gel in this composite mesh provided MSCs a 3-dimensional environment. It has been found that MSCs in 3-dimensional culture increase the expression of scleraxis, a marker gene of tenogenic [26]. Alginate solution polymerizes quickly by adding calcium. This offers the convenience to incorporate MSCs to the composite mesh in a setting of operation room.

When MSCs were delivered to the defects between Achilles tendon and gastrocnemius/soleus with MSC-loaded mesh, tendon repair was improved at days 6 and 14 as indicated by the histological scores. However, it was only at day 6, this improvement was statistically significant. The effect of MSCs on the early stage of tendon healing was also shown in an animal study of Achilles rupture, where MSC injections increased the mechanical strength of the repaired tendons at an early stage (1–2 weeks) of tissue healing but not at the late stage (after 3 weeks) [28]. At day 6, the improvement of tendon repair in M + S group was supported with dense, parallel collagen bundles and increased extracellular matrix, but reduced vascularity was the main contributing factor. In terms of the exact role of MSCs played in the process of tendon repair in M + S group, however, it remains for future investigation. MSCs are regenerative and there are MSCs in tendon [29]. In a variety of tendon/ligament repair models, applications of MSCs improve the healing and mechanical property of the tendon [17-19]. It is highly possible that, in the current study, locally delivered MSCs went tenogenic differentiation and participated in regeneration of neo-tendon. Unlike MSC osteogenesis, chondrogenesis and adipogenesis that can be induced with standardized protocols, the condition of MSC tenogenic differentiation is still under development. It has been found, however, that tendon itself promotes tenogenic differentiation of MSCs [30,31]. In the current study, MSCs were delivered to tendon defects, which may present MSCs an optimal environment for tenogenesis. In addition, these MSCs might secrete a variety of cytokines and growth factors that suppress the local immune reaction, inhibit fibrosis (scar formation) and stimulate tissue-intrinsic reparative or stem cells for regeneration [10].

It is interesting to note that the cellularity was not a contributing factor distinguished the M + S group from others at day 6. This suggests that, at least, the implantation of MSC-loaded mesh did not induce significant inflammatory response locally. It is believed that inflammation causes scar formation and inevitably affect the function of the repaired tendon [32].

The composition and organization of collagen in the repairing tissue greatly influence tendon strength. In natural tendon healing, there are structural and functional deficiencies that lead to inferior mechanical strength to healthy tendon. In fact, the repaired tendon does not achieve the normal failure force mechanically in years [33]. At day 14, the MSC-loaded mesh repaired tendon with significantly more collagen formation and much better organized collagen bundles than the mesh only. It suggests MSC implantation has the potential to improve the mechanical strength of repaired tendon. This is in line with other studies that applied MSCs to tendon repair. In most of the studies, an improved mechanical property of the repaired tendon stands out after applications of MSCs [20,28]. It is noteworthy that post-transcriptional remodeling of collagen fibrils by matrix metalloproteinases also contributes to the improved mechanical property of the MSC-mediated tendon repair [26].

Types I collagen is predominant in the extracellular matrix of normal tendon. M + S group was the only one that showed a significantly increased area of deposition of type I collagen from day 6 to day 14. There is potential that the increased type I collagen in the matrix may improve the strength of the repaired tendon in the M + S group. During tendon repair, type III collagen is increased initially and persistent in scaring tissue [34,35]. In the present study, type III collagen was indifference among the three groups in either staining area or intensity. When the ratio of type III collagen over type I collagen was considered, the staining area ratio in the M + S group was significantly greater than in the Sut group. The addition of MSCs seemed to interfere with the process of tendon healing and changed the composition of extracellular matrix. However, the staining area ratio was gradually decreased over the time in both M + S and M groups, but increased in Sut group. Although these trends were not statistically significant in the current study, different repairing materials (i.e. surgical mesh vs. suture) and the consequently mechanical environment might influence the proportion of types III and I collagen in the repaired tendons [26].

The initial inflammatory, proliferative phases of tendon repair involve in coordinated and complex molecular and cellular events, but last for a relatively short period [1]. MSCs implanted into the ruptured tendons most likely interfere with the biology of early tendon repair. This study was primarily focused on the early events after repairing tendon defects with MSC-loaded surgical mesh. The limitations of this study include the repaired tendons were not assessed with biomechanical properties and the animals were followed up for only a short period of time. Immunohistochemistry provides useful information about the distribution patterns and localization of types I and III collagen. However, it is not an ideal method for quantification of types I and III collagen in the repaired tendons.

## Conclusion

In summary, a surgical mesh was modified with polymer scaffold and hydrogel for transplantation of MSCs for tendon repair. The implantation of MSC-loaded mesh was able to deliver MSCs locally and enhanced early tendon healing. Although the density of delivered MSCs and the functional recovery of the repaired tendons are to be evaluated in future studies, this study demonstrated that MSC application influences the process of tendon repair fundamentally [36]. The results are significant for the care and surgery of tendon ruptures, as gaining strength early will benefit to the functional recovery of repaired tendons.

## Competing interests

The authors declare that they have no competing interests.

## Authors’ contributions

LCS, NG and JN designed the study and performed animal surgery. MT, JD, JM and JK conducted histological analyses. LCS, MT and ZZ drafted the manuscript. All authors read and approved the manuscript.
